# Hodgkin Lymphoma Mimicking Lumbar Spine Tuberculosis

**DOI:** 10.1155/2022/5298960

**Published:** 2022-02-25

**Authors:** H. Julien Djossou, Mohamed Ahmed Ghassem, Hamza Toufik, Mohamed Oukabli, Ahmed Bezza, Lahsen Achemlal

**Affiliations:** ^1^Rheumatology Department, Military Hospital Mohammed V, Rabat, Morocco; ^2^Anatomy Pathology Department, Military Hospital Mohammed V, Rabat, Morocco

## Abstract

**Introduction:**

The clinical manifestations of Hodgkin lymphoma (HL) can closely mimic spine and lymph node tuberculosis (TB). *Case Description*. A 48-year-old man was initially treated for retroperitoneal lymph nodes TB, and this diagnosis was made without bacteriological and histopathological confirmation. After four months of regular therapy for TB, he did not improve and was admitted to our department for lumbar spine pain. We first made diagnosis of tuberculous spondylodiscitis, and anti-TB treatment was strengthened. But, after three weeks of hospitalization, his condition worsened clinically with onset of swelling of the left supraclavicular lymph node. So, after surgical excision and anatomopathological examination of the lymph node, the diagnosis of nodular sclerosis classic Hodgkin lymphoma was made. He was treated by chemotherapy, and his condition improved significantly after the first 2 cycles of chemotherapy.

**Conclusion:**

Repeated investigations may be helpful in establishing a correct diagnosis and starting an effective treatment in this highly curable disease.

## 1. Introduction

Apart from muscle, ligament, or soft-tissue damage, low back pain can result from an inflammatory or infectious pathology or even from a primary or metastatic vertebral tumor. Thus, Hodgkin Lymphoma (HL) and tuberculosis (TB) are two causes of low back pain, and their clinical and radiological signs can closely mimic each other. The problem occurs when after a first biopsy, the anatomopathological examination finds a granulomatous lesion. This is the case we want to talk about in this study.

## 2. Case Report

A 48-year-old man was admitted to our department for low back pain. He had a history of diabetes treated with oral antidiabetics for eight years and a history of active chronic smoking evaluated at 30 pack-years without any history of TB. Four months before his hospitalization in our department, he presented with digestive transit disorders and diffuse abdominal pain associated with insidious inflammatory low back pain. These signs evolved in a context of deterioration of the general condition with night sweats and weight loss estimated at 36 Kg in 6 months without fever. So, he was initially admitted to the digestive diseases department where the thoracoabdominopelvic CT scan objectified retroperitoneal lymphadenopathies without mediastinal and pulmonary involvement. Thus, a surgical excision biopsy with anatomopathological examination of the retroperitoneal lymph nodes showed rare epithelioid granulomas without caseous necrosis. However, after a negative search for acid-fast bacilli in the sputum and a positive interferon-gamma release assay, the diagnosis of retroperitoneal lymph node TB was made and they decided to treat the patient with anti-TB drugs (regimen: isoniazid, rifampin, ethambutol, and pyrazinamide for 2 months (intensive phase) followed by isoniazid and rifampin for 4 months (continuation phase)).

**Figure 1:**
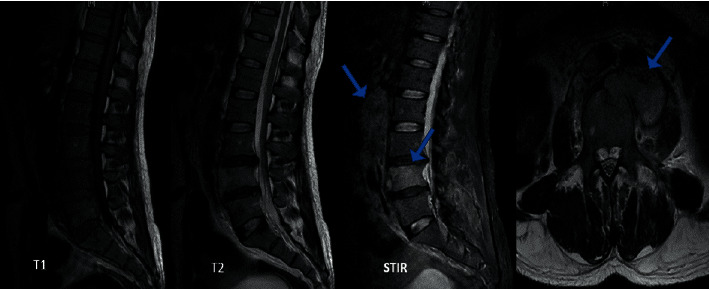
Lumbar spine and pelvis magnetic resonance imaging.

After four months of anti-TB treatment, the patient was admitted to our department for worsening of his low back pain radiating to the buttocks and becoming permanent with insomnia. At the initial physical examination, he had a lumbar spine syndrome without neurologic signs and without superficial lymphadenopathy or hepatosplenomegaly. X-ray examinations of the lumbar spine and pelvis were without abnormality. Magnetic resonance imaging (MRI) showed an involvement of the L4 vertebral body realizing hyposignal in T1 and hypersignal in T2 and STIR, a disc involvement secondary to the rupture of the lower cortex of L4, a collection of the paravertebral soft parts facing and the left psoas measuring 46 mm × 32 mm, with retroperitoneal lymphadenopathies (Figure 1). Given the clinical context of retroperitoneal lymph node TB, we first considered the case as vertebral tuberculous with psoas abscess. We strengthened the anti-TB treatment, and we contacted the neurosurgery department for a surgical drainage of the psoas abscess. However, psoas abscess surgery did not take place, because after three weeks of hospitalization in our department, the patient presented with a soft swelling of the left inguinal region identified on ultrasound as multiple lymphadenopathies. He also presented with a left supraclavicular lymphadenopathy and a worsening of the biological inflammatory syndrome compared to admission values (three weeks earlier). So, C-reactive protein ranged from 124.2 to 137.4 mg/L, and erythrocyte sedimentation rate (ESR) ranged from 90 to 100 mm; at the hemogram, lymphopenia ranged from 1000 to 600 lymphocytes/*µ*L, and normochromic normocytic anemia ranged from 11.3 to 9 g/dl, and the platelet count remained normal.

**Figure 2:**
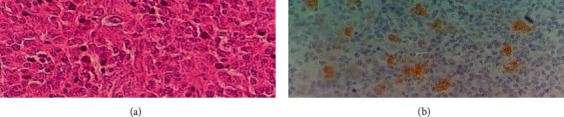
Anatomopathological and immunohistochemical examinations of the left supraclavicular lymph node. (a) Large Hodgkin-like cells on a dense background (HEx40); (b) large cells are CD30 positive (x40).

**Figure 3:**
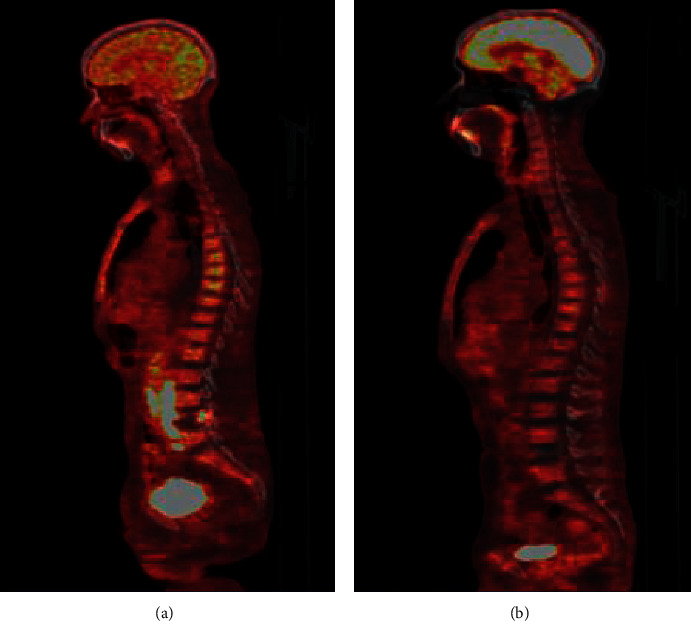
PET scan. (a) Before chemotherapy; (b) after 2 cycles of chemotherapy.

A surgical excision biopsy of the left supraclavicular lymph node was performed, and the anatomopathological examination concluded with the diagnosis of nodular sclerosis classic HL (Figure 2). Immunohistochemical study showed positive expression of anti-CD15 and anti-CD30 antibodies by tumor cells. Human immunodeficiency virus (HIV) serology was negative, and lactate dehydrogenase (LDH) value was normal. The PET scan objectified diffuse and disseminated pathological hypermetabolic foci in almost all of the explored skeleton, in the cervical, thoracic, and abdominopelvic lymph nodes, in the spleen, and in the left gluteal muscles (Figure 3(a)). The patient was, therefore, treated in the department of hematology by the chemotherapy BEACOPP regimen (bleomycin, etoposide, doxorubicin hydrochloride (Adriamycin), cyclophosphamide, vincristine (Oncovin), procarbazine, and prednisone). After the first 2 cycles of chemotherapy, he improved clinically significantly, and the control PET scan showed a marked reduction in pathological hypermetabolic foci (Figure 3(b)).

## 3. Discussion

Our study has a double interest. First, it reveals spinal involvement at the early stage of HL. Bone involvement in HL is usually observed in the late stage, but very rarely in the early stage. It occurs in 10 to 15% of patients and preferentially concerns the axial skeleton and the proximal part of the long bones [1]. Bone lesions occur through hematogenous spread or direct spread from the contiguous involved lymph node.

The second and most important particularity of our study is to reveal the diagnostic difficulty between HL and TB, especially when a first pathological examination reveals a granulomatous lesion.

In the literature, cases of concomitant HL with TB have been reported, and a cause-and-effect relationship has been suggested [2–5]. However, despite the description of the association between HL and TB in several studies, we strongly reject the diagnosis of TB in our case. In fact, even though we were in an endemic area for TB, the diagnosis of TB in our patient has not been based on histological and/or bacteriological evidence. Not only that, although our patient initially received regular treatment for TB, he did not improve but worsened clinically. The case that stood out to us was about a 63-year-old woman who was admitted for suspected reactivation of lymph node tuberculosis previously treated successfully and diagnosed one year earlier [6]. The clinical signs consisted of worsening of the patient's general condition with cervical lymphadenopathy and dyspnea. Under the assumption of reactivation of lymph node tuberculosis, the patient was initially treated with an extended tuberculostatic therapy. However, the disease continued to worsen. Another lymph node biopsy was performed, revealing HL of mixed-cellularity type with a partly histiocytic necrotizing, partly tuberculoid reaction, but the biopsy was negative for acid-fast bacilli. Thus, after the chemotherapy ABVD protocol (Adriamycin, Bleomycin, Vinblastine, and Dacarbazine), her clinical condition rapidly improved. Another study reported the case of an 81-year-old man who presented with cervical/supraclavicular lymphadenopathy in a context of fever, night sweats, and weight loss [7]. The revised lymph node histopathological examination revealed nodular sclerosis classical HL associated with abundant TB-mimicking granulomatous reaction.

In front of lymph node or vertebral involvement suggestive of TB, when the anatomopathological examination shows only a granulomatous lesion without caseous necrosis and negative acid-fast staining, the clinician should evoke a diagnosis of lymphoma. A study reported the case of a 30-year-old man, a chronic smoker who was initially treated for a presumptive impression of TB for 6 months without improvement [8]. At the initial diagnosis, there was hilar lymphadenopathy and a few very small (<0.5 cm) abdominal and axillary lymph nodes. Histopathological examination of axillary lymph nodes showed a few epithelioid cell granulomas without necrosis or acid-fast bacilli. The correct diagnosis of HL was finally established when a bone marrow biopsy (for fever and pancytopenia) revealed the typical neoplastic Reed–Sternberg (RS) cells.

Even if the clinician suspects a widespread disease almost endemic, he cannot take shortcuts to validate the diagnosis. The diagnostic trap between TB and HL lies in the similarities in clinical course, imaging, and laboratory tests. When there is a strong suspicion of TB, molecular detection of mycobacteria should be used. In a study evaluating the performance of molecular detection and identification of the *Mycobacterium tuberculosis* complex and other clinically important nontuberculous mycobacteria, the overall sensitivity, specificity, and positive and negative predictive values were 52.9, 100, 100, and 97.6% for extrapulmonary samples, respectively [9]. When the *Mycobacterium* cannot be detected, the differential diagnosis between TB and HL is based primarily on RS cell morphology and immunohistochemical profile.

In conclusion, our case suggests that clinicians strongly discuss the diagnosis of lymphoma, even when TB seems clinically evident but anti-TB treatment has failed. Repeated investigations, especially biopsy and histopathological examination, may be helpful in establishing a correct diagnosis.

## References

[1] Borg M. F., Chowdhury A. D., Bhoopal S., Benjamin C. S. (1993). Bone involvement in Hodgkin’s disease. *Australasian Radiology*.

[2] Reddy R. C., Mathew M., Parameswaran A., Narasimhan R. (2014). A case of concomitant Hodgkin’s lymphoma with tuberculosis. *Lung India*.

[3] Centkowski P., Sawczuk-Chabin J., Prochorec M., Warzocha K. (2005). Hodgkin’s lymphoma and tuberculosis coexistence in cervical lymph nodes. *Leukemia and Lymphoma*.

[4] Mahajan K., Gupta G., Singh D. P., Mahajan A. (2016). Simultaneous occurrence of Hodgkin’s disease and tubercular lymphadenitis in the same cervical lymph node: a rare presentation. *BMJ Case Reports*.

[5] Boilève A., Kuhnowski F., Cassou-Mounat T., Jehanno N., Kirova Y. (2020). Hodkgin lymphoma concomitant of tuberculosis, a therapeutic challenge for multidisciplinary management. *Cancer Radiotherapie: journal de la Societe francaise de radiotherapie oncologique*.

[6] Audebert F., Schneidewind A., Hartmann P., Kullmann F., Schölmerich J. (2006). Lymphknotentuberkulose als erstmanifestation eines morbus Hodgkin. *Medizinische Klinik*.

[7] Szumera Ciećkiewicz A., Prochorec-Sobieszek M., Lech-Marańda E. (2014). Hodgkin’s lymphoma mimicking tuberculosis in cervical lymph nodes. *Polish Journal of Pathology*.

[8] Badyal R. K., Sharma P., Prakash G., Malhotra P., Varma N. (2014). Hodgkin lymphoma masquerading as tuberculosis in a young chronic smoker. *Indian Journal of Hematology and Blood Transfusion*.

[9] Bicmen C., Gunduz A. T., Coskun M., Senol G., Cirak A. K., Ozsoz A. (2011). Molecular detection and identification of *Mycobacterium tuberculosis* complex and four clinically important nontuberculous mycobacterial species in smear-negative clinical samples by the genotype mycobacteria direct test. *Journal of Clinical Microbiology*.

